# Matrix Metalloproteinase 1: Role in Sarcoma Biology

**DOI:** 10.1371/journal.pone.0014250

**Published:** 2010-12-08

**Authors:** Muhammad Umar Jawad, Nandor Garamszegi, Susanna P. Garamszegi, Mayrin Correa-Medina, Juan A. Diez, Rong Wen, Sean P. Scully

**Affiliations:** 1 Department of Orthopedics, University of Miami Hospital, University of Miami Miller School of Medicine, Miami, Florida, United States of America; 2 Department of Ophthalmology, Bascom Palmer Eyes Institute, McKnight Vision Centre, University of Miami Miller School of Medicine, Miami, Florida, United States of America; 3 Diabetes Research Institute, University of Miami Miller School of Medicine, Miami, Florida, United States of America; Virginia Commonwealth University, United States of America

## Abstract

In carcinomas stromal cells participate in cancer progression by producing proteases such as MMPs. The expression MMP1 is a prognostic factor in human chondrosarcoma, however the role in tumor progression is unknown. Laser capture microdissection and In Situ hybridization were used to determine cellular origin of MMP1 in human sarcomas. A xenogenic model of tumor progression was then used and mice were divided in two groups: each harboring either the control or a stably MMP1 silenced cell line. Animals were sacrificed; the neovascularization, primary tumor volumes, and metastatic burden were assessed. LCM and RNA-ISH analysis revealed MMP1 expression was predominantly localized to the tumor cells in all samples of sarcoma (p = 0.05). The percentage lung metastatic volume at 5 weeks (p = 0.08) and number of spontaneous deaths secondary to systemic tumor burden were lower in MMP1 silenced cell bearing mice. Interestingly, this group also demonstrated a larger primary tumor size (p<0.04) and increased angiogenesis (p<0.01). These findings were found to be consistent when experiment was repeated using a second independent MMP1 silencing sequence. Prior clinical trials employing MMP1 inhibitors failed because of a poor understanding of the role of MMPs in tumor progression. The current findings indicating tumor cell production of MMP1 by sarcoma cells is novel and highlights the fundamental differences in MMP biology between carcinomas and sarcomas. The results also emphasize the complex roles of MMP in tumor progression of sarcomas. Not only does metastasis seem to be affected by MMP1 silencing, but also local tumor growth and angiogenesis are affected inversely.

## Introduction

The process of sarcoma metastasis is an event in which mesenchymal tumor cells escape confines of local disease control measures and threaten the life of the host. The metastatic process thus is a very attractive target for novel therapies but a fundamental understanding of the process is necessary to design effective therapies. The mechanisms underlying the metastatic cascade of sarcomas are largely unknown and may differ significantly from that of carcinomas in which there is a preliminary understanding. Many cellular and molecular elements of the tumor microenvironment have emerged as attractive targets for therapeutic strategies among carcinomas. One such putative target, metalloproteinases, have been implicated in many processes involved in tumor progression including anti-angiogenic therapies [1].

Although MMPs have been implicated in a variety of diseases such as arthritis, atherosclerosis etc., it was evidence of their role in cancer progression that lead to attempts at therapeutic application [2]. Several broad spectrum synthetic MMP inhibitors were put into clinical trials; results of which have largely been disappointing [3]. There have been numerous studies presenting conflicting evidence of a pro-tumorigenic versus protective roles of various MMPs [4], [5]. This highlights the need for an improved understanding of specific roles for different MMPs in tumor progression that can lead to a more targeted and hopefully successful therapy in future.

Most of the *in vitro/in vivo* studies and clinical trials exploring MMP inhibition in cancer were on carcinomas which are malignancies of epithelial origin. In these malignancies, the development of a tumor requires support from the surrounding host stromal tissue, also referred to as the tumor microenvironment [6]. Carcinoma-associated fibroblasts, leukocytes, bone marrow-derived cells, blood and lymphatic vascular endothelial cells present within the tumor microenvironment contribute to tumor progression [7]. The dynamic and reciprocal interactions between tumor and host cells orchestrate events critical to tumor progression. Tumor cells have been shown to induce MMP production in surrounding stromal and inflammatory cells of mesenchymal origin such as fibroblasts, macrophages and mast cells. Numerous studies utilizing RNA *in situ* hybridization to evaluate MMP expression in human tumor tissue revealed that most MMPs are predominantly expressed by stromal cells which are of mesenchymal origin [8]. These studies were performed on carcinomas arising in organs such as lung, breast, head and neck, prostate, bladder, and colon [9]. Recently, Gupta et al. have reported MMP1 as a member of lung metastatic gene signature (LMS) for breast carcinoma [10]. They also proposed sub-categorizing the genes involved in metastasis and recognized MMP1 as a metastatic progression gene: a gene that has dual functions in mediating primary tumorigenesis and metastatic colonization, for a specific breast cancer cell-line model [11].

In malignancies of mesenchymal origin, little is known about the role of MMPs in tumor progression. Previously, we reported the prognostic significance of MMP1 gene expression in patients with chondrosarcoma [12]. Subsequent studies demonstrated the correlation of MMP1 silencing by antisense oligonucleotides and shRNA with reduced invasive potential of sarcoma cells *in vitro* [13], [14], [15], [16], [17], [18]. We hypothesize that the majority of MMP production can be attributed to tumor cells in sarcomas in contrast to mesenchymal stromal cells in carcinomas. Determining this difference is critical if inhibition of MMP activity is to be entertained as a potential antitumor therapy in these tumors.

Based on our earlier *in vitro* findings we hypothesized that the effects of stable MMP1 silencing would lead to decreased metastasis *in vivo*. In order to determine the target cell population for MMP1 silencing, we carried out an expressional analysis of MMP1 in human chondrosarcoma specimens by both laser capture microdissection and *in situ* hybridization. These two complementary lines of evidence indicate that sarcoma cells serve as the primary source of MMP1 in this tumor. Subsequently, the potential of MMP1 as a therapeutic target in human sarcoma was tested in an orthotopic xenogenic model using shRNA technique to stably silence MMP1 in human sarcoma cells. The results demonstrate that MMP1 silencing was associated with a trend of decreased rate of pulmonary metastasis but also increases in primary tumor volume and vascularization. Whether these represent direct effects by decreasing pericellular collagen degradation or indirect effects will require further study [19].

## Results

### Cellular Origin of MMP1 in human sarcoma samples

**Figure 1:**
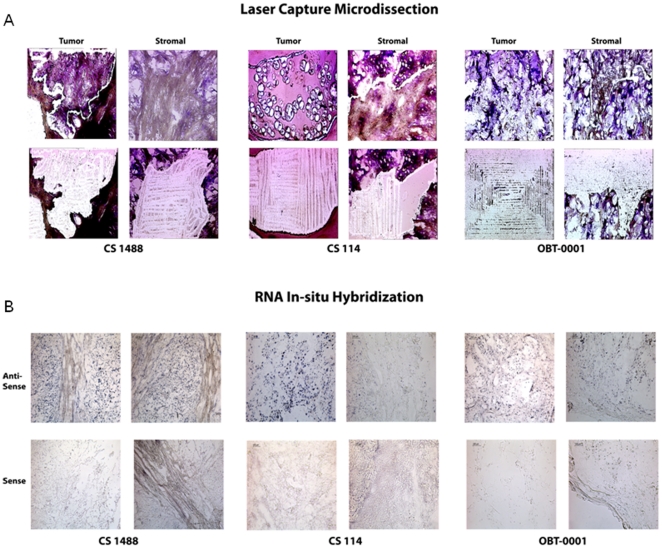
Cellular Origin of MMP1. Figure 1 A: Laser Capture Micro-dissection pictures at 20× magnification for CS1488, CS 114 and OBT-0001, Figure 1 B: In-situ RNA Hybridization results for CS 1488, CS 114 and OBT-0001. Black staining reveals the MMP expression in the tissue. The stained part in figure 1b with vacuolization represents the tumor cells and the unstained fibrous part represents the stromal cells.

The cellular origin of human chondrosarcoma was evaluated in sections of surgical isolates using both laser capture microdissection and in situ hybridization. Quantitative RT- PCR was used to quantitate the MMP1 mRNA in human chondrosarcoma cells and in adjacent stromal cells following isolation by laser capture microdissection (Figure 1; Panel A). Each cell population was collected and processed separately and MMP1 gene expression analysis was performed using the ABI Taqman gene expression assays. Concurrent analysis of TIMP1 gene expression, the naturally occurring inhibitor for MMP1, and two housekeeping genes: a structural gene, 18S, and an expressional gene, B2M, were performed for quality assurance and normalization of gene expression [20]. The level of gene expression for each sequence from both host and tumor cell populations are shown in table 1. MMP1 gene expression was detectable largely in the tumor cell population (p = 0.05). The range of difference in expression of MMP1 between tumor cells and stromal cells after normalization ranged from 4 -fold to a 100-fold difference. There was minimal MMP1 gene expression detected in host stromal cells. The control genes (including TIMP1 and the two housekeeping genes) demonstrated comparable expression in tumor and stromal cells for each sample indicating mRNA recovery and quantitation were similar in tumor and stromal cells (Table 1). This corroborates previous studies and that the observed difference in expression of MMP1 between host and tumor cells is genuine; and the populations of cells collected for analysis from the tumor and stromal aspects of the tumor were comparable.

**Table 1:** Results of QPCR Analysis for CS 1488, CS 114 and OBT-0001.

Sample		18S	TIMP1	MMP1	B2M	MMP1:18S
**CS 1148**						
	**Tumor**	4.41E-05	9.06E-08	1.96E-06	1.88E-07	4.44E-02
	**Tumor**	9.07E-05	1.35E-07	6.03E-06	2.31E-07	6.65E-02
	**Stromal**	8.75E-04	3.84E-07	4.22E-07	6.54E-06	4.82E-04
	**Stromal**	3.76E-05	4.64E-07	3.19E-07	2.05E-05	8.48E-03
**CS 114**						
	**Tumor**	5.41E-03	3.84E-06	2.10E-06	1.35E-05	3.88E-04
	**Tumor**	9.06E-04	1.02E-06	4.79E-07	2.17E-06	5.29E-04
	**Stromal**	1.60E-03	8.09E-07	1.73E-07	2.28E-06	1.08E-04
	**Stromal**	1.50E-03	8.97E-07	2.15E-07	2.96E-07	1.43E-04
**OBT-0001**						
	**Tumor**	3.58E-05	1.77E-07	6.27E-07	2.97E-07	1.75E-02
	**Tumor**	1.11E-04	3.00E-07	1.96E-07	2.26E-07	1.77E-03
	**Stromal**	4.32E-05	2.00E-07	4.28E-08	2.06E-07	9.91E-04
	**Stromal**	3.21E-05	1.90E-07	Undetermined	2.01E-07	Undetermined

Messenger RNA In-Situ Hybridization (ISH) was used as an independent means of ascertaining the cellular origin of MMP1 gene expression in human sarcoma tissue. Corresponding photomicrographs for each tumor sample are shown in (Figure 1; Panel B). In-situ hybridization with an anti-sense probe demonstrates that MMP1 gene expression is detectable only in tumor cells and not in surrounding host stromal cells. A sense probe was used as a control in parallel sections under the same hybridization conditions. These did not reveal any staining in the tumor or in the stromal part for all three samples indicating the specificity of the hybridization. This result was consistent for all three human chondrosarcoma samples.

### MMP1 Silencing of Human Sarcoma cells

**Figure 2:**
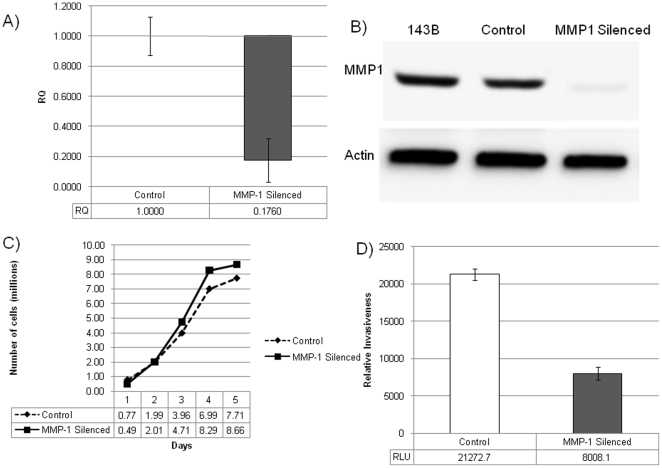
Results of MMP1 Silencing. Figure 2A) Relative quantity (RQ) of MMP1 in the stable clones of 143B cell-line prepared. MMP1 Silenced is 98% down-regulated as compared to control. Control  = 1.00±0.13; MMP1 Silenced = 0.18±0.14. Figure 2B) displays the Western Blot analysis for MMP1 production by the stable clones of 143B cell-line. MMP1 Silenced clone shows down-regulation at the level of protein as compared to control. Figure 2C) displays *in vitro* proliferation assay showing growth of control and MMP1 silenced clones over 5 days. There is an approximately 14% increased growth displayed by MMP1 silenced clone in the log-phase of growth. Difference in mean growth over 5 days is statistically insignificant (p = 0.804). Figure 2D) displays results for the in vitro invasion assay measured in Relative Light Units (RLU). Control = 21272.7±790; MMP1 silenced = 8008.1±874.5 RLUs (p = 1.54E-09).

To determine the involvement of MMP1 in chondrosarcoma metastasis, MMP1 gene expression was stably silenced using shRNA approach. QPCR was used to determine the relative quantities of MMP1 gene expression in the two stably shRNA transfected clones and are displayed in Figure 2A. There was a 98% reduction of MMP1 gene expression of in silenced cells when compared with the control cells (scrambled shRNA sequence). Western blotting verified that the MMP1 gene expression silencing resulted in a reduced protein level (Figure 2B). There were no significant changes in gene expression of other collagenases, specifically MMP8 and MMP13 indicating that the shRNA silencing was specific for MMP1. We repeated our experiment to confirm our findings using a second shRNA sequence: T6-7. When compared to scrambled sequence, there was a 70% (as opposed to 96%) reduction of MMP1 gene expression (data not shown).

### In Vitro Cell Proliferation and Invasion

In an attempt to determine the effect of MMP1 gene expression silencing *in vitro* on cell proliferation and invasion, assays of these parameters in control and silenced cell clones were determined, Figure 2 C and D. The difference in mean proliferation of the two clones over 5 days was not statistically significant (p = 0.80). A Boyden chamber employing a type 1 collagen surface was used to assess *in vitro* invasion over 48 hrs. Control cells were determined to be 2.66× more invasive than MMP1 silenced cells, p<0.001 (Figure 2D).

### Primary Tumor Growth

**Figure 3:**
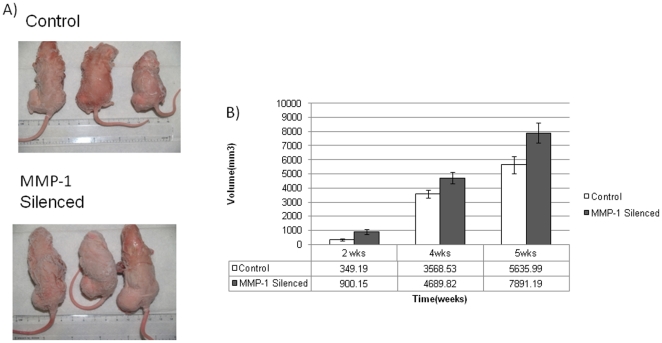
Primary tumor size. Tumor volumes in ‘control’ and ‘MMP1 silenced’ groups at 5 week intervals are shown in Figure 3A. Figure 3B displays the histograms showing mean volume of tumor (mm^3^) for a single mouse in control and MMP1 silenced groups at corresponding time points. Difference in tumor volumes at 2, 4 and 5 weeks is significant with p-values of 0.023, 0.036 and 0.026.

The effect of MMP1 gene expression silencing was then evaluated in vivo in a xenogenic model of metastasis. Primary tumor volume was assessed in tumor bearing mice (*n* = 70) at 2, 4 and 5 weeks (figure 3A). A graphical representation of mean tumor volume (mm^3^) for a single mouse over time and the differences among the two groups of mice bearing control and silenced clones of 143B cell-line are depicted (Figure 3B). Tumors were visibly larger in mice bearing the MMP1 silenced clone and continued to show increased growth over time as depicted in the histogram. The differences in the mean tumor volume in two groups were statistically significant at 2, 4, and 5 weeks, (p<.04) (control = 349±79; MMP1 = 900±180 mm^3^ 2 wks, control  = 3568±277; MMP1 = 4690±404 mm^3^ 4 wks, and control  = 5636±615; MMP1 = 7891±716 mm^3^ 5 wks respectively). Further analysis revealed that MMP1 silenced group had a constant rate of tumor growth over 5 weeks whereas mice bearing control group showed a plateau in tumor growth over time. There was no difference in survival between mice with control and MMP1 silenced tumor clones. The experiment was repeated using the T6-7 clone and the primary tumor growth followed similar trends. Mice bearing T6-7 clone grew bigger tumors at 2 and 5 weeks (p<0.05) (supplementary Table S1).

### Primary Tumor Vascularity

**Figure 4:**
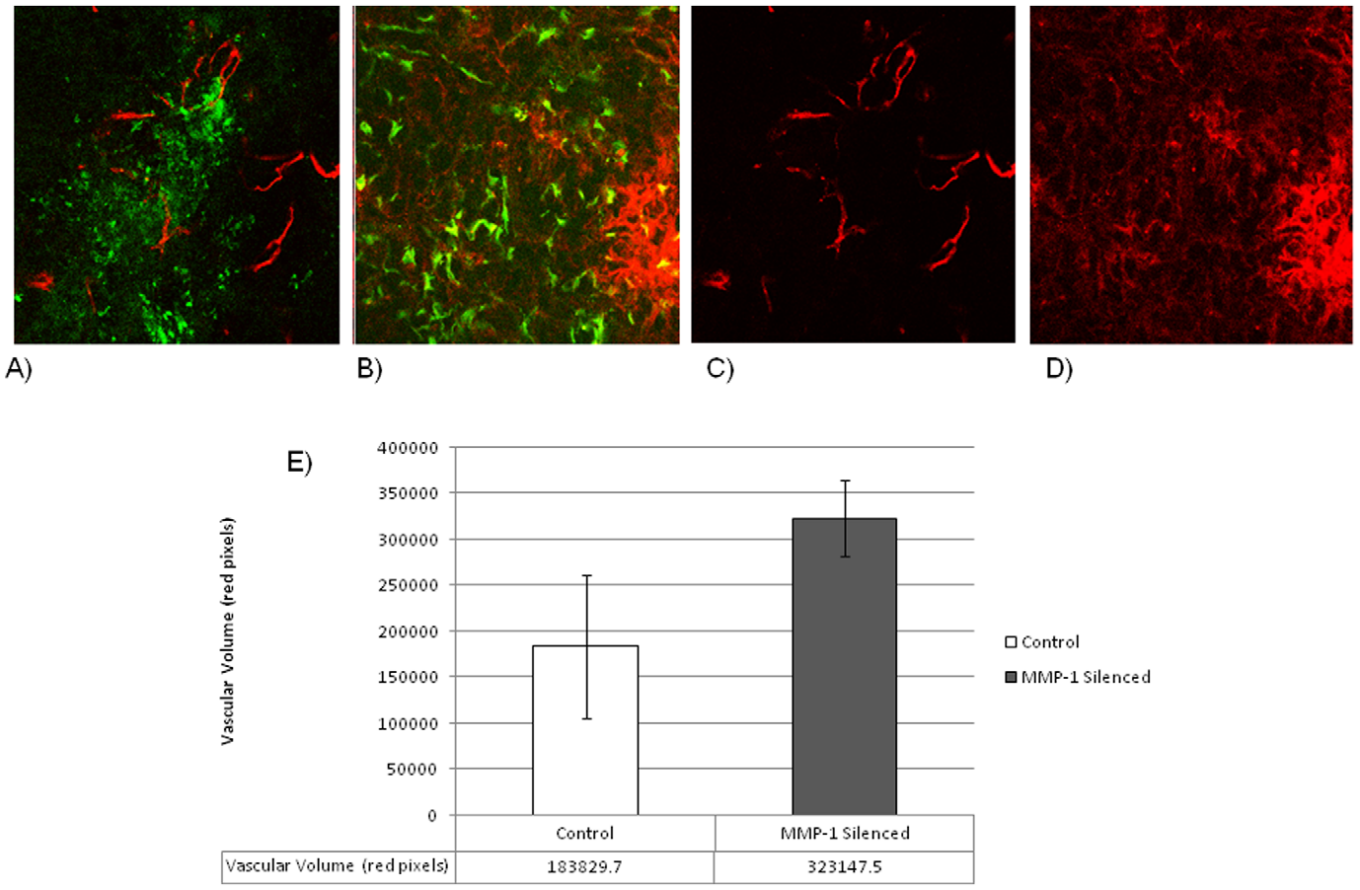
Primary tumor Vascularity. Confocal Images showing primary tumor from a) control group and b) MMP1 Silenced group. Images c) and d) display only the blood vessels from a) and b). Figure 4E shows histograms representing average vascular volume (number of red pixels) for a single field of vision (at 25×) in tumors harbored by control (183829.7±78022) and MMP1 Silenced (323147.5±41141) groups. Difference in mean vascular volume is significant with p-value = 0.01.

In an attempt to understand the effects of MMP1 gene silencing on primary tumor growth, tumor vascularity was assessed. Tumor vasculature was labeled with a fluorescent dye and then imaged with confocal microscopy. Photomicrographs from the two groups of mice are shown in Figure 4. The primary tumor in the control group consisting of GFP labeled sarcoma cells and Di-I stained red blood vessels are depicted in Figure 3A. The image demonstrates new tumor associated vessels. Corresponding images from MMP1 silenced group of mice are depicted in Figure 4B. Distinct neoangiogenic sprouts can be appreciated and highlighted in the supplemental video S1, corresponding to Figure 4B. In figure 4C & D, the vascularity of the tumors is measured by isolation of red fluorescence signal intensity for both the control and silenced cells. The tumor containing MMP1 silenced cells demonstrates increased vascularity and increased number small vessels approaching the tumor cells.

Analysis of vascularity revealed a statistically significant increase in mean vascular volume per unit volume of tumor for MMP1 silenced group (321233±41140 threshold pixels) as compared to control (184283±78021 threshold pixels) group (p = 0.01) (Figure 4E). This trend was consistent when the experiment was performed again using T6-7 clone. The mean vascularity for T-67 clone was almost twice as high as for control clone (supplemental Table S1).

### Pulmonary Burden

**Figure 5:**
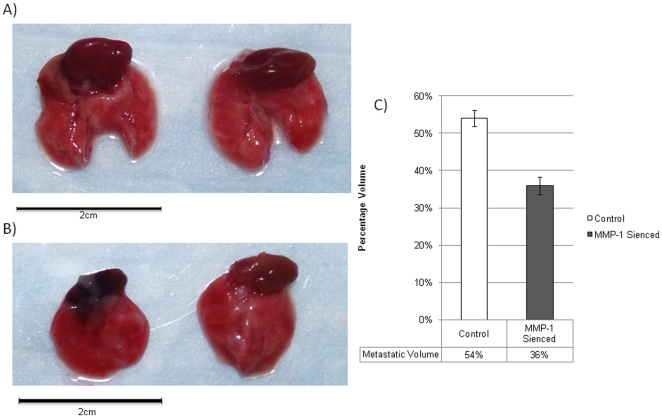
Metastasis in Lungs. Lungs showing metastatic areas from control (Figure 5A) and MMP1 silenced groups (Figure 5B) are shown. Figure 5C shows histogram representing average percentage of total lung volume affected by metastasis at 5 weeks for control (54±17.6%) and MMP1 Silenced (36±23.7%) groups. Difference in mean percentage area affected by metastasis is not significantly different, p = 0.08.

The effect of MMP1 silencing on metastasis was determined following necropsy at five weeks when the lungs were isolated. Figure 5 shows a digital photograph of two pairs of lungs from A) control and B) MMP1 silenced groups clearly showing areas of gross metastasis. An estimation of percentage of total lung volume affected by metastasis revealed a higher percentage of volume affected in control group (54%±2.20) as compared to MMP1 silenced group (36%±2), p = 0.08. This information is graphically depicted in Figure 5C. Once again, this trend is also consistent with T-67 clone although the difference was further reduced between T-67 and control as shown in the supplementary file.

### Characterization of Isolated sarcoma cells from mice

**Figure 6:**
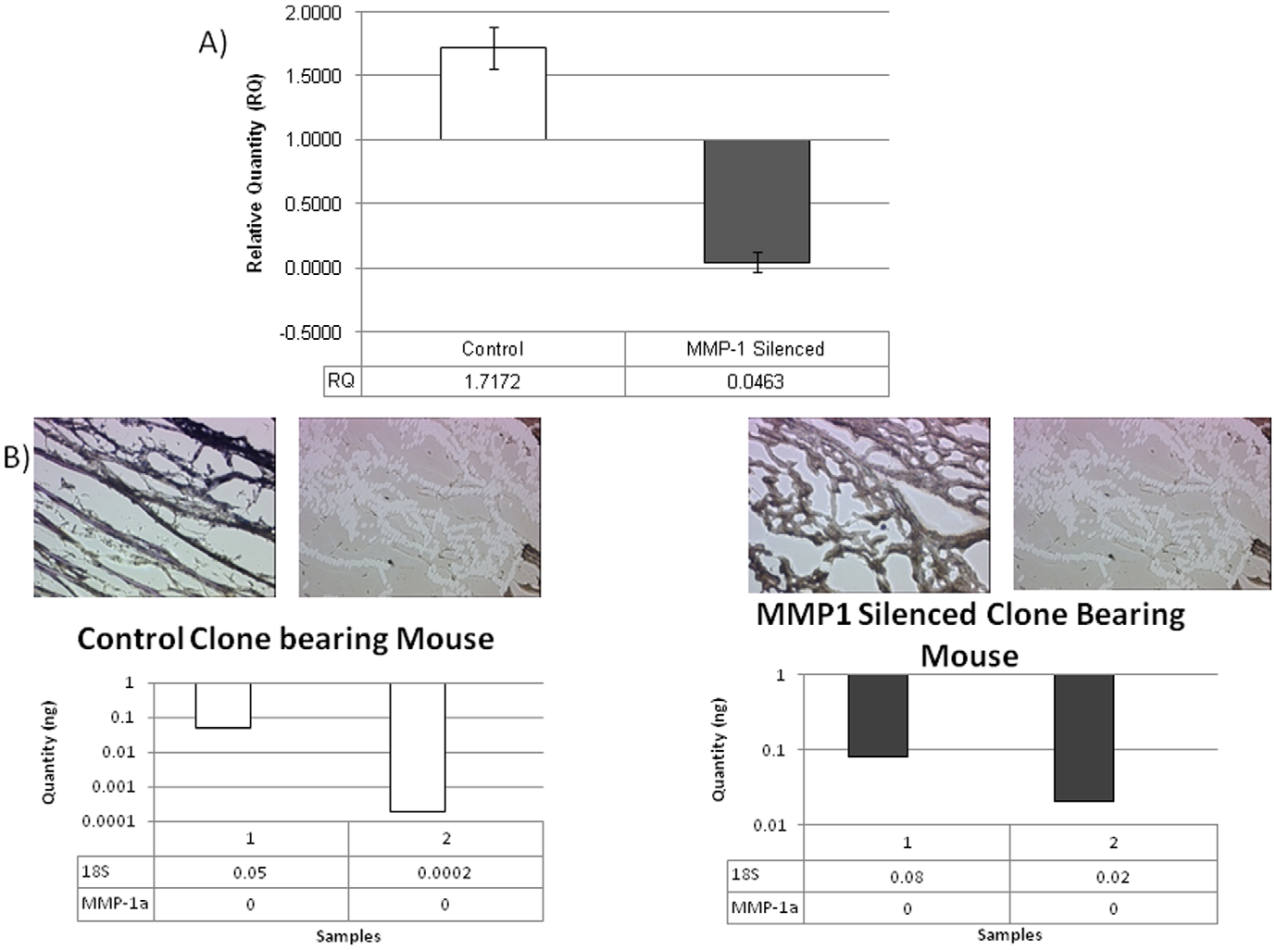
Assessment of stablization of MMP Silencing. Figure 6A shows the Relative quantity (RQ) of MMP1 in the control and MMP1 silenced clones isolated from mouse lungs at necropsy. MMP1 Silenced clone is 95% down-regulated as compared to control. Control  = 1.72±0.17; MMP1 Silenced = 0.05±0.08. Figure 6B shows MMP1a production by peri-tumoral murine stromal tissue as assessed with laser capture microdissection and QPCR.

Stability of MMP1 over time was assessed by examining gene expression post necropsy in pulmonary metastasis. Figure 6A shows the relative level of MMP1 expression in cells recovered from the control group and the silenced group. The relative level of MMP1 expression in control and silenced isolated cells (97% down) was similar to the original extent of MMP1 silencing prior to implantation indicating stability of the shRNA effect through the in vivo tumor growth and metastasis. Furthermore, peri-tumoral MMP1a & MMP1b production by murine stromal tissue was assessed with laser capture microdissection and QPCR, figure 6B. Murine stromal cells in both control and silenced cells did not produce murine MMP1 at detectable levels. Murine MMP 13 contribution was also negligible (data not shown). Hence MMP1 was present only in human tumor cells without an induced contribution from the surrounding murine cells.

Lack of murine tissue positive for MMP1a, MMP1b and MMP13 in the vicinity of tumor limited us to include a positive control for the above mentioned experiment. However, the proper functioning of murine primers was established in other experiments carried out on a murine breast cancer cell line.

## Discussion

Prior to 1990, most findings regarding MMP1 expression were derived from analysis of cell lines and a few immunohistochemical studies of human tumors [21]. These results were consistent with the concept that MMPs proteins were mainly expressed by cancer cells. However, *in situ* localization studies to evaluate MMP gene expression directly in human cancer specimens dramatically modified this concept [22]. A majority of studies determining the origin of MMPs in carcinomas were based only on *in situ* mRNA hybridization techniques [23], [24]. Indeed, most MMPs were found to be predominantly expressed by stromal cells of mesenchymal origin in human carcinomas, but not in the cancer cells of epithelial origin [25]. This was regarded as a fundamental change in the MMP biology in a neoplastic microenvironment of carcinomas and questioned the validity of MMP silencing in studies of carcinoma metastasis [25].

The majority of human malignancies are of epithelial origin (i.e. carcinomas) and thus little, if any, attention has been given to the origin of MMPs in malignancies of mesenchymal origin (i.e. sarcomas). However, many sentinel discoveries of tumor biology have arisen from studies of these tumors [26]. The current report is the first in-depth analysis of role of MMP1 in sarcoma biology. The study of the microenvironment of sarcomas reveals an interesting situation where cancer cells of mesenchymal origin are surrounded by stromal cells of the same origin. Understanding the relationship of host and tumor cells in mesenchymal malignancies is likely to provide new insights into tumor progression that may be of broader applicability beyond this group of infrequent malignancies.

Earlier results from our laboratory indicate a prognostic significance of MMP1 expression in disease specific survival of patients with chondrosarcoma [12]. The current study extends the understanding of the role of MMP1 in sarcoma biology by localizing the synthesis to tumor cells. Two complementary and independent approaches were used in the current study to address this question. Laser capture microdissection has been a very sensitive technique to distinguish tumor and stromal gene expression for a number of malignancies including breast, prostate, pancreatic, colon and gastric cancers [27], [28]. Similarly mRNA-ISH technique has been used to determine gene expression in a number of tumors including giant cell tumors of bone [29]. Interestingly, both experimental approaches indicate that MMP1 is primarily expressed in human sarcoma cells as and not the surrounding stromal cells. This result is in sharp contrast to what has been reported in the literature for carcinomas [28]. Furthermore in the examination of xenogenic metastasis where murine and human MMP1 can be distinguished, it is clear that sarcomas do not induce MMP1 expression in surrounding stromal tissue.

This finding has several implications on both experimental, as well as therapeutic aspects. MMPs have been widely implicated as a therapeutic target for antitumor therapy [10]. Xenogenic models have been primarily used to delineate the role of MMPs in tumor carcinoma progression. RNA interference techniques to silence MMPs have been used in numerous carcinoma cell lines and a melanoma cell line [10], [30]. These cell lines were subsequently injected in animals to investigate the role of MMP1 silencing on tumor progression. These models have an inherent flaw because the major source of MMPs in carcinoma biology is the stromal cells which remain MMP1 competent after tumor cell silencing. Our finding encouraged us to investigate the role of MMP1 in a xenogenic sarcoma model that appears to be more appropriate model for investigation of the role of MMPs in tumor biology, as there is minimal contribution of MMPs from stromal cells.

The current study is unique and comprehensive for determining the cellular origin of MMP1 in sarcoma samples, carrying out the stable silencing of target cells and following disease progression in a xenogenic murine model of sarcoma. It demonstrates that MMP1 silencing results in increased local tumor growth and increased vascularity of the primary tumor but less systemic disease burden in a xenogenic murine model of human sarcoma. This is the first report of localized pro-tumorigenic effects of MMP1 silencing and more importantly the first report of opposing effects on local growth versus systemic tumor burden of MMP1 in an animal model of metastasis.

Martin and Matrisian recently reviewed protective roles of MMPs in tumor progression [5]. MMP3 has been shown to decrease initial growth rates of squamous cell carcinoma in wild type mice compared to knockouts [31]. Another study reported decreased development of mammary tumors in transgenic mice expressing MMP3 [32]. MMP12 expression has been implicated in increased number of well-differentiated tumors with better outcomes [33]. Another collagenase, MMP8 when silenced resulted in decreased latency period and increased number of papillomas [34].

Role of MMP1 has been well documented in the establishment of lung metastasis in breast cancer [35] as well for establishment of primary lung cancer [36]. Gupta et al. suggested a role of MMP1 in vascular remodeling co-opted for sequential steps in lung metastasis [10]. Current results support these findings and provide evidence for a pro-metastatic role of MMP1 in a xenogenic murine model of human sarcoma. Higher rates of pulmonary tumor burden in control group resulted in a higher incidence of spontaneous deaths. Opposing effects on local tumor growth and systemic tumor burden have historically been reported for stromelysin-3 (MMP11) [37]. The difference in pulmonary tumor burden and survival in two groups of mice in the current study are not statistically significant. It is very important to interpret these results recognizing that 1) mice dying spontaneously (control = 6; silenced = 2) could not be assessed for pulmonary burden of disease because of interval tissue necrosis and 2) the experimental plan was truncated because of primary tumor burden being greater than 10% body weight (5 weeks) than originally planned (8-12 weeks) per IACUC stipulations. Mice dying spontaneously had a higher systemic disease burden as compared to the rest of the cohort. A higher number of spontaneous deaths negatively skewed the pulmonary burden in control group as compared to MMP1 silenced group. While the study was adequately powered from the outset, this may represent a beta statistical error.

Patients with pancreatic and small-cell lung cancer showed significantly poorer outcome with and MMP inhibitor therapy leading to early termination of the trial [3]. Subsequently reports have emerged over the past decade that challenged the dogma of proteases being exclusively tumor promoting potentially explaining these clinical results. Merchan et al. reported anti-angiogenic and anti-proliferative effects mediated by uPA's protease activity [38]. The observation that MMP1 silencing is associated with increased primary tumor volume is novel and is in contrast to what has been observed in breast cancer where MMP1 silenced tumors demonstrated reduced primary tumor size.[39] The possible underlying mechanisms for increased primary tumor size in sarcoma is unclear and may be secondary to an underlying catabolism of anabolic factors or other mitogenic signaling molecules by MMP1. An alternative mechanism could be secondary to increased vascular perfusion [19].

Pro- and anti-angiogenic roles of different MMPs in tumor angiogenesis have been well documented in literature [40]. MMP1 has been implicated in release of bFGF and VEGF from the extracellular matrix to promote angiogenesis [41], [42]. It has also been implicated in targeting endothelial proteinase-activated receptor 1 and thus activating endothelial cells [43]. Brinckerhoff et al. recently showed that RNAi mediated inhibition of MMP1 lead to reduction of angiogenesis in murine models of melanoma and breast cancer [4], [30]. We herein report an increase in tumoral angiogenesis as a result of MMP1 silencing. Several other MMPs including MMP 2, 3, 7, 9, 12 have been shown to perform both pro- and anti-angiogenic roles [40]. Mechanisms for anti-angiogenic effects include cleavage of FGFR1 and uPAR, the release and subsequent binding of PEX domain to α_v_β_3_ on MMP degradation, and formation of potent angiogenesis inhibitor peptides. One of such peptides is endostatin, generated by proteolytic cleavage of C-terminal of type XVIII collagen α1 chain. Another collagenase MMP13 has been implicated in its production of endostatin [44]. Since MMP1 and MMP13 share the same collagen cleavage point, it may be that MMP1 is manifesting its anti-angiogenic effects through this mechanism.

The method described is a new method for visualizing the vascularity in tumor bed. But we feel it is more accurate as compared to traditional immune-histochemistry with CD 31 or CD 34 antibodies. In this method, vascularity is assessed in three dimensions in a 50 micron thick section and from each tumor 5 representative areas were imaged, ensuring adequate representation. Immunohistochemistry is limited to two dimensions only and can be misleading especially when investigating a highly vascular tumor bed since vessels can be running along the plane of section or the same vessel can be crossing the plane of section at multiple points, potentially giving a false assessment. Thus, we believe our method, although new, provides a better assessment of vascularity. We would also like to suggest a comparison of this method with the traditional immunohistochemistry method in future.

This study highlights that there are fundamental differences in terms of MMP biology between sarcomas and carcinomas. Not only the cellular origin of MMP differs, but MMP1 silencing affects the disease progression in xenogenic models in different ways. In addition to the hypothesized pro-metastatic roles, the current study also identified additional effects on vascularity and primary tumor growth [11]. Repeating the experiment using a a second shRNA sequence and subsequent MMP1 silenced tumor cell clone allows verifies that findings reported are real and not a result of clonal variation or artifact. A gene acting as metastasis progression gene for a breast cancer model may act as a metastasis initiation gene or a metastasis virulence gene for a sarcoma model. There are several potential limitations of the current study including limited mechanistic insight to these novel observations. The precise cellular and molecular mechanisms of MMP1 silenced cells leading to increase in local tumor growth; angiogenesis and a lower pulmonary burden are currently being pursued.

## Materials and Methods

Three human chondrosarcoma samples were obtained from the tumor bank at the Sylvester Comprehensive Cancer Center following Institutional Review Board (IRB) approval. Informed written consent was taken from all the patients after detailed written and verbal description of the contents of consent. This procedure was reviewed and approved by the University of Miami ethics committee for research.

### Laser Capture Microdissection

A laser capture microdissection technique was used to separate the tumor cells and the surrounding stroma. A 5 micron cryosection in OCT was obtained using Leica^R^ digital cryostat at -20°C and sections were mounted on Director^R^ slides that employ unique Laser Induced Forward Transfer (LIFT), a non-contact microdissection technology. Approximately 500 cells were collected per sample. LCM Hisogene^R^ Arcturus^R^ kit was used to stain and dehydrate the sections and mRNA isolation was carried out using an Arcturus^R^ Pico-pure^R^ RNA Isolation kit. RT reaction was carried out using ABI oligo-dT probes (ABI Taqman Gene Expression Assays^R^ Mm473485_m1) and QPCR was carried out using ABI Real time Fast QPCR. Gene expression levels for 18S, TIMP1, MMP1 and B2M were investigated for each sample. For each tumor sample, 2 different populations of tumor and stromal cells were microscopically dissected (Figure 1). Quantitative PCR was performed on each of the tumor and stromal cell populations collected for all three chondrosarcoma samples (Table 1). Five different samples of tumor and stromal cells were dissected from the entire section for each sample corresponding to a tumor and a stromal sample in Table 1 for each of three chondrosarcoma samples. A Paired Student t-test was used to compare the mean of MMP1:18S for tumor and stromal cell populations.

### Messenger RNA FISH

*In situ* hybridization using a DIG –labeled RNA probe for human MMP1 (Exiqon) was performed as described [45]. Briefly, a total of 17 nM of DIG-labeled probe was diluted into 100 µl of hybridization buffer, applied to the slides and allowed to hybridize at 70°C overnight. Slides were then washed for 1 h at 70°C in 0.2× SSC solution (Ambion-Applied Biosystems, CA) and incubated with alkaline phosphatase-conjugated sheep anti-DIG antibody(1∶2500, Roche) overnight at 4°C. Alkaline phosphatase reaction was carried out in PVA with 200 µl of MgCl_2_ 1 M and 140 µl of NBT/BCIP stock (Roche). Sense strand probe (Exiqon) was used as a negative control.

### Cell Culture and Reagents

The 143B sarcoma cell line was purchased from ATCC and cells were grown in Dulbecco's modified Eagle's medium (DMEM Invitrogen #10-0117CV) containing 10% fetal bovine serum (FBS) and antibiotics in incubator set to 37°C with 5% CO_2_.

### Preparation of 143B Stable Clones

The stable clones were generated by pHuSH 29mer shRNA constructs against MMP1 (#TR311450 RNAi targeting vector system OriGene Rockville, MD) as described [46]. We were provided with four different sh RNA sequences and we used two different sequences to generate T6-4 and its respective control clone and the second independent clone T6-7 and its respective control. Briefly, clones selected by puromycin (Invitrogen, 1.0 µg/ml). Individual colonies were isolated and expanded in selection media containing the antibiotics until passage was transferred into p100 plates. Then clones cultured further for two passages and screened for gene expression changes by QPCR. The screening process was repeated multiple times to test expressional stability. After verification of stable expression profiles, a control line (V9) and an MMP1 RNAi silenced clone (T6-4) were selected. These clones were further transformed with pEGFP-N1 fluorescent reporter construct with FuGENE HD (# 04709705001, Roche Corp.) to aid their identification (Clontech Laboratories Inc. #6077-1 Mountain View, CA). Expressional profiles were tested again to verify any changes in MMP1 RNAi down-regulation levels, and then the established lines were stored frozen. The expression of MMP1 mRNA relative to control was 0.016±0.123. The second independent clone T6-7 and its respective control clone were generated using the same protocol, but a different sequence of sh RNA supplied by OriGene Rockville, MD.

### RNA Purification and Relative PCR

Total RNA was extracted from by using the RNeasy Mini kit (Qiagen Corp. US #74104) according to the manufacturer protocol. RT reaction was generated (Applied Biosystems # 4368813) and relative PCR was performed (Applied Biosystems Taqman^R^ Gene Expression Assay Hs00233958_m1). The data was then analyzed by ΔΔCt method with the supplied software, and according to the manufacturer's protocol.

### Western Blotting and Image acquisition

Cells were lysed in RIPA buffer (150 mM NaCl, 1.0% IGEPAL® CA-630, 0.5% sodium deoxycholate, 0.1% SDS, 50 mM Tris, pH 8.0. # R0278 Sigma Saint Louis MO), containing protease inhibitor cocktail (Roche # 04693124001). The samples were normalized for protein with ND-1000 (NanoDrop Technologies Inc., Wilmington DE). For western blotting, 125 µg protein per lane were analyzed with primary antibodies incubated overnight at 4°C, followed by secondary HRP conjugated antibodies for 2 hours at room temperature. Bands were detected with SuperSignal West Pico ECL detection kit (# 34080, Pierce, Rockford IL) on UVP Biospectrum Digital Imaging system (UVP Inc. Upland CA).

### In Vitro Proliferation Assay

The *in vitro* cellular growth capacity was measured over 5 day period. Each day a triplicate set was trypsinized, cells counted on hematocytometer, and discarded. The logarithmic growth phase (∼between days 3 and 4) was used to determine growth speed in culture (ΔX/ΔT) which expressed then as % difference relative to control.

### In Vitro Invasion Assay

The QCM™ 96-well collagen-based cell invasion assay Chemicon International (#ECM556) was used. After initial cell number normalization, 4.0×10^4^ cells/well were seeded onto the plate (9 parallels with 3 blank controls). The feeder wells contained the same media +/- FBS (10%) to determine the effects of natural metalloproteinase inhibitors, and incubated overnight. The following day the plates were processed and measured with 480/520 nm filter set on Berthold Mithras LB 970 plate reader.

### Xenogenic Model of Metastasis

A xenogenic murine model of human sarcoma was used to assess the effect on metastasis [14]. Four week old 70 SCID *nu/nu* mice were divided into two groups of 35 mice each. One million cells in a 10 micro liter volume were orthotopically implanted in the left tibial metadiaphysis using a 27 gauge needle in two groups of 35 animals each. Control group was implanted with GFP +143B (V9) cell line and an MMP1 silenced group was implanted with GFP + shRNA 143B (T6-4) cell line.

Sample size was calculated using the formula for one sided test. The MMP1 silenced clone was expected to decrease the metastatic potential of this cell line with a mean of 0.5 and range of 0-2. Power 95%, alpha = 0.01, beta = 0.05. [14] Sample size was then modified upward considering the rate of engraftment failure known to occur. Institutional IACUC approval from University of Miami (Institutional Animal Care and Use Committee University of Miami) for this project was obtained (Approval Number 07-251). We repeated the experiment using a second independent MMP1 shRNA sequence and subsequent stably transfected clone of tumor cells. For that experiment we used 6 mice in each group and sacrificed the animals at 2 and 5 weeks.

### Di-I Staining

Di-I staining was carried out using the protocol described by Wen et al. Briefly, fluorescent carbocyanine dye Di-I solution (1, 1′-Dioctadecyl-3, 3, 3′, 3′-tetramethylindocarbo-cyanine perchlorate; Catalog # 42364, Sigma-Aldrich, St. Louis, MO) and 4% paraformaldehyde (20 ml in 0.1 M phosphate buffer, pH 7.4). was injected into the left ventricle. By the end of procedure, the paws, tail and the ears turn pink indicating a successful systemic perfusion of Di-I [47].

### Tumor Measurement

Local tumor volume was measured using the technique described by Luu et al. [14]. Volume =  (L) (W) (L+W) (0.2618), where 0.2618 is the constant of proportionality.

An average of volume measurements by the two observers was used as the final value.

### Vascular Measurement

Local tumors were isolated after sacrificing the animal and immediately embedded in OCT, snap frozen in liquid nitrogen and stored. Tumor vascularity was measured using confocal microscopy on a 50 micron thick frozen section using a 25×/0.8 µA water immersion lens on the Zeiss LSM 510/UV confocal microscope. Five different areas, each having fluorescently labeled 143B cells and Di-I stained blood vessels, were imaged and z-series for entire thickness of tissue was determined. This procedure was repeated for 5 different cryosections and evaluated by two observers [48]. A 3D reconstruction (LSM Image Browser) of each image was used to calculate the Di-I stained area using the Metamorph Imaging system 5.0^R^ (Universal Imaging Corp., Downingtown, PA.). A total of 7 mice from each group were used for this estimation.

### Pulmonary Burden

An *ex-vivo* estimation of pulmonary burden was made using digital images of lung tissue and delineation with ImageJ^R^ v1.4 (NIH). Metastatic volume was determined using Metamorph Imaging system 5.0^R^ (Universal Imaging Corp., Downingtown, PA.). Metastatic area is presented as a percentage of total lung volume. An *ex-vivo* estimation of pulmonary burden was also made using the Xenogen IVIS (In-vivo imaging system). Region of Interest measurements were made using the specific excitation and emission wavelengths for the flurophore (GFP: FITC) used to label the 143B cell line. Some, but not all of metastatic volume on visual inspection corresponded with fluorescent area. Thus we used visual delineation of metastatic area for the purpose of analysis.

### Isolation of sarcoma cells from mouse tissue

Sarcoma cells were isolated out of the mouse primary tumor as well as lung metastasis. The tumors were surgically excised and homogenized with a surgical blade to small sizes that fit through the 25 ml pipette, then transferred to a 50 ml conical tube and incubated ∼ three hours at 37°C with occasional gentle vortexing. Following the incubation the mixture was filtered through Falcon Cell Strainer (#352350 with 70 µm pore size Becton Dickinson Labware, Franklin Lakes NJ) and 1 ml aliquots were dispersed into p100 plates containing 143B culturing media completed with puromycin selection. The isolated cells were re-grown in culture and characterized for expression of MMP1.

### Laser Micro-Dissection to assess the contribution of host MMP1

Host MMP1 production was assessed to determine if tumor cell MMP1 silencing was compensated for by surrounding stromal cells. Murine stromal cells were dissected out from the 5 micron thick frozen section of primary tumor using laser capture micro-dissection technique. Arcturus^R^ Histo-Gene^R^ RNA Isolation kit was used to isolate RNA according to manufacturer's protocol. Expression analysis (QPCR) on 2 samples from control group and 4 samples from silenced MMP1 silenced mice was carried out (ABI Taqman Gene Expression Assays^R^ Mm473485_m1).

### Statistical Analysis

Data are presented as means with standard error of mean (SEM). Differences in means from the *in vitro* and *in vivo* experiments were compared by using the Student's *t* test. Differences were considered significant at p<0.05. All statistical tests for analysis of outcomes were two-tailed. Kaplan-Meier and Log Rank methods are used for analysis of survival and differences were considered significant at p<0.05.

## Supporting Information

Table S1 Data from the second experiment showing comparison of second independent MMP1 silenced clone T6-7 and its respective control group.
(0.03 MB XLS)

Video S1 (0.51 MB MP4)
